# Cerebrospinal fluid proteome associations with neuropsychiatric symptoms in Alzheimer's disease

**DOI:** 10.1002/alz70856_103643

**Published:** 2025-12-24

**Authors:** Miriam Rabl, Pyry Helkkula, Willem Lucas Hartog, Wiesje M. van der Flier, Yolande A.L. Pijnenburg, Charlotte E. Teunissen, Pieter Jelle Visser, Betty M. Tijms, Julius Popp

**Affiliations:** ^1^ Psychiatric University Hospital Zurich and University of Zurich, Zurich, Zurich, Switzerland; ^2^ Alzheimer Center Amsterdam, Amsterdam UMC location VUmc, Amsterdam, North‐Holland, Netherlands; ^3^ Alzheimer Center, Department of Neurology, Amsterdam UMC, Vrije Universiteit Amsterdam, Amsterdam Neuroscience, Amsterdam, Netherlands; ^4^ Alzheimer Center Amsterdam, Neurology, Vrije Universiteit Amsterdam, Amsterdam UMC location VUmc, Amsterdam, Amsterdam, Netherlands; ^5^ Alzheimer Center Amsterdam, Neurology, Vrije Universiteit Amsterdam, Amsterdam UMC location VUmc, Amsterdam, Netherlands; ^6^ Neurochemistry Laboratory, Amsterdam UMC, Amsterdam, Netherlands; ^7^ Department of Neurobiology, Care Sciences and Society, Division of Neurogeriatrics, Karolinska Institutet, Stockholm, Sweden; ^8^ Alzheimer Center Limburg, Mental Health and Neuroscience Research Institute, Maastricht University, Maastricht, Netherlands; ^9^ Alzheimer Center Amsterdam, Department of Neurology, Vrije Universiteit Amsterdam, Amsterdam UMC location VUmc, Amsterdam, Netherlands; ^10^ Department of Psychiatry, Old Age Psychiatry Service, Lausanne University Hospital, Lausanne, Switzerland

## Abstract

**Background:**

Neuropsychiatric symptoms (NPS) are common in older people and may represent early symptoms of dementia, in particular in Alzheimer's disease (AD). Little is known about the underlying pathological mechanisms. We used untargeted proteomics to investigate CNS molecular pathway alterations related to the presence and severity of NPS in older people.

**Method:**

Individuals with normal cognition (NC), mild cognitive impairment (MCI) or mild AD dementia from the Amsterdam Dementia Cohort with available untargeted mass spectrometry‐based cerebrospinal fluid (CSF) proteomics data were included. A total of 1962 proteins was considered for analysis. NPS were measured using the Neuropsychiatric Inventory‐Questionnaire (NPI‐Q), an informant‐based questionnaire assessing twelve behavioral symptoms. Linear and logistic regression models were applied to study relationships of protein CSF levels (predictor) with the total NPI‐Q severity score (range 0‐36, outcome) and presence of individual NPS (outcome). Interaction terms of amyloid status and CSF protein levels on NPS were tested to study if results were specific to AD. Pathway enrichment analysis using the Reactome and Gene Ontology databases were used to characterize the identified proteins.

**Result:**

A total of 362 individuals (120 with NC, 78 with MCI, 164 with AD dementia) with mean age of 64.0±7.7 years and 41.7% women were included. Men presented with more severe NPS (NPI‐Q score 5.0 vs. 4.0 in women, *p* = 0.011) and more frequently experienced symptoms of apathy (57.3% vs. 43.0%), irritability (56.9% vs. 40.4%), agitation (20.4% vs. 9.9%), and disinhibition (20.4% vs. 8.6%) than women (*p* <0.01). Preliminary findings revealed twelve CSF proteins associated with NPI‐Q severity scores (*p* <0.01), independent of amyloid status. We identified distinct protein alterations related to the presence of single symptoms. Notably, we found no overlap of proteins associated (*p* <0.01) with the three most frequent symptoms, i.e., apathy (51.4%, 13 proteins), irritability (50.0%, 19 proteins), and depression (35.6%, 25 proteins), respectively. Next, we will perform pathway enrichment analysis to identify the specific biological pathways involved in NPS.

**Conclusion:**

Using untargeted CSF proteomics, we identified different protein profiles related to overall NPS severity and presence of individual symptoms. First results suggest distinct pathological mechanisms involved in NPS, which may represent specific intervention targets.

